# Transcriptomic profiling and quantitative high-throughput (qHTS) drug screening of *CDH1* deficient hereditary diffuse gastric cancer (HDGC) cells identify treatment leads for familial gastric cancer

**DOI:** 10.1186/s12967-017-1197-5

**Published:** 2017-05-01

**Authors:** Ina Chen, Lesley Mathews-Greiner, Dandan Li, Abisola Abisoye-Ogunniyan, Satyajit Ray, Yansong Bian, Vivek Shukla, Xiaohu Zhang, Raj Guha, Craig Thomas, Berkley Gryder, Athina Zacharia, Joal D. Beane, Sarangan Ravichandran, Marc Ferrer, Udo Rudloff

**Affiliations:** 1 Thoracic and Gastrointestinal Oncology Branch, National Cancer Institute, National Institutes for Health, CCR 4 West/4-3740, 10 Center Drive, Bethesda, MD 20892-0001 USA; 2 Washington University School of Medicine, St. Louis, KY USA; 3 Division of Preclinical Innovation, National Center for Advancing Translational Sciences, National Institutes of Health, Rockville, MD USA; 4 Department of Biology and Center for Cancer Research, Tuskegee University, Tuskegee, AL USA; 5 Surgery Branch, NCI, NIH, Bethesda, MD USA; 6 Genetics Branch, NCI, NIH, Bethesda, MD USA; 7 Indiana University School of Medicine, Indianapolis, IN USA; 8 Advanced Biomedical Computing Center, Frederick National Laboratory for Cancer Research, Leidos Biomedical Research Inc., Frederick, MD USA

**Keywords:** Hereditary diffuse gastric cancer (HDGC), High throughput drug screening, Therapeutic leads, c.1380delA CDH1 gastric cancer cells, Differential gene expression profiling

## Abstract

**Background:**

Patients with hereditary diffuse gastric cancer (HDGC), a cancer predisposition syndrome associated with germline mutations of the CDH1 (E-cadherin) gene, have few effective treatment options. Despite marked differences in natural history, histopathology, and genetic profile to patients afflicted by sporadic gastric cancer, patients with HDGC receive, in large, identical systemic regimens. The lack of a robust preclinical in vitro system suitable for effective drug screening has been one of the obstacles to date which has hampered therapeutic advances in this rare disease.

**Methods:**

In order to identify therapeutic leads selective for the HDGC subtype of gastric cancer, we compared gene expression profiles and drug phenotype derived from an oncology library of 1912 compounds between gastric cancer cells established from a patient with metastatic HDGC harboring a c.1380delA CDH1 germline variant and sporadic gastric cancer cells.

**Results:**

Unsupervised hierarchical cluster analysis shows select gene expression alterations in c.1380delA CDH1 SB.mhdgc-1 cells compared to a panel of sporadic gastric cancer cell lines with enrichment of ERK1–ERK2 (extracellular signal regulated kinase) and IP3 (inositol trisphosphate)/DAG (diacylglycerol) signaling as the top networks in c.1380delA SB.mhdgc-1 cells. Intracellular phosphatidylinositol intermediaries were increased upon direct measure in c.1380delA CDH1 SB.mhdgc-1 cells. Differential high-throughput drug screening of c.1380delA CDH1 SB.mhdgc-1 versus sporadic gastric cancer cells identified several compound classes with enriched activity in c.1380 CDH1 SB.mhdgc-1 cells including mTOR (Mammalian Target Of Rapamycin), MEK (Mitogen-Activated Protein Kinase), c-Src kinase, FAK (Focal Adhesion Kinase), PKC (Protein Kinase C), or TOPO2 (Topoisomerase II) inhibitors. Upon additional drug response testing, dual PI3K (Phosphatidylinositol 3-Kinase)/mTOR and topoisomerase 2A inhibitors displayed up to >100-fold increased activity in hereditary c.1380delA CDH1 gastric cancer cells inducing apoptosis most effectively in cells with deficient CDH1 function.

**Conclusion:**

Integrated pharmacological and transcriptomic profiling of hereditary diffuse gastric cancer cells with a loss-of-function c.1380delA CDH1 mutation implies various pharmacological vulnerabilities selective to CDH1-deficient familial gastric cancer cells and suggests novel treatment leads for future preclinical and clinical treatment studies of familial gastric cancer.

**Electronic supplementary material:**

The online version of this article (doi:10.1186/s12967-017-1197-5) contains supplementary material, which is available to authorized users.

## Background

Hereditary diffuse gastric cancer (HDGC) is an autosomal dominant cancer susceptibility syndrome due to germline mutations within the E-cadherin (CDH1) gene locus cadherin (CDH1; NM_004360) [1, 2]. HDGC is clinically defined by the familial occurrence of early-onset diffuse gastric cancer (DGC) and lobular breast cancer [2–4]. Of the 26,370 cases of gastric cancer expected to be diagnosed in the United States in 2016, 5 to 10 percent arise in a familiar context and about 34–45% of these are due to CDH1 germline mutations [4, 5]. CDH1 germline mutations are most commonly truncating CDH1 variants affecting the extracellular domains of the E-cadherin gene leading to loss of CDH1 expression, followed by missense and splice site variants and, infrequently, large genomic deletions [2]. CDH1 mutations are heterozygous and dispersed across the 16 exons of CDH1 [6]. Hypermethylation of the CDH1 promotor of the unaffected wild type allele and loss of heterozygosity are considered the most common second hit mechanisms of CDH1 inactivation and loss [7]. Male CDH1 mutation carriers have by the age 80 a cumulative incidence of gastric cancer of 70%, female mutation carriers a risk of 56% of gastric and 42% for lobular breast cancer [2]. Risk-reducing gastrectomy and breast MRI surveillance is currently advised for all patients with CDH1 germline gene mutations [4].

The natural history and clinical course of HDGC patients differs significantly from patients afflicted by sporadic gastric cancer: HDGC harbors a unique early stage (T1a) characterized by foci of intramucosal signet-ring cell carcinoma confined to the lamina propria [4, 8]. The number of these foci of early invasive cells may exceed one hundred and are thought to originate as an early event from displaced daughter cells of neoplastic cells with reduced CDH1 expression at the upper neck of gastric glands which have lost their physiological epithelial localization due to loss of cytoskeletal organization, cell plasticity and cell polarity [8, 9]. Further invasion beyond the gastric mucosa is associated with poor differentiation, Src kinase activation, and epithelial-to-mesenchymal transformation [10]. While the natural history of these early intramucosal lesions is incompletely understood, it is thought that they might be relatively indolent and that there is, unlike in sporadic gastric cancer, a latency period of possibly many years towards further progression [4]. Clinically, patients with advanced familial gastric cancer and a genetic loss of CDH1 have a worse clinical outcome compared to patients with epigenetic silencing or no CDH1 alteration [11].

Despite these molecular, histopathological, and clinical differences, patients with HDGC receive, in large, the same, largely ineffective cytotoxic chemotherapy regimens as sporadic gastric cancer patients. In contrast, recent differential gene expression, synthetic lethality, and high throughput drug screening studies in isogenic E-cadherin deficient (−/−) breast MCF10A cells identified several select genetic and pharmacological vulnerabilities in CDH1(−/−) mutant cells [12]. While some of the observed activity profiles in CDH1-deficient cells, like select sensitivities to Src kinase in the CDH1(−/−) mutant MCF10A isoform, were in line with early signaling perturbations observed in T1a cancers of gastrectomy specimens of HDGC patients, other signaling aberrations observed in clinical specimens later in the transition of invasion beyond the gastric mucosa, like FAK and STAT3 kinase activation, were not found to be associated with select drug sensitivities in the MCF10A CDH1(−/−) in vitro system [10, 12]. As the MCF10A CDH1(−/−) isogenic system captures predominantly early vulnerabilities with predominantly chemopreventative translational value, it is thus not known if the synthetic lethalities and drug sensitivities discovered to selectively occur in the CDH1(−/−) mutants capture vulnerabilities of E-cadherin deficient gastric cancers in a more evolved stage which might harbor greater therapeutic value.

To develop new therapeutic strategies for HDGC patients, we compared drug sensitivity profiles derived from a dose response quantitative high throughput drug (qHTS) screens of 1912 oncology compounds between c.del1380A CDH1 gastric cancer cells derived from a HDGC patient, and wild type CDH1 gastric cancer cells derived from a sporadic gastric cancer patient. We combined the differential pharmacological responses with gene expression profiles between c.del1380A CDH1 and a cohort of sporadic gastric cancer cells. Increased ERK1/2 and phosphatidylinositol 4,5-bisphosphate (PIP2)-mediated signaling in c.1380delA CDH1 cells was accompanied by increased sensitivities to mTOR, AKT, MEK, protein kinase C (PKC) and topoisomerase II (TOPO2) signaling inhibition. We also observed increased sensitivities to compounds belonging to ALK (Anaplastic Lymphoma receptor tyrosine Kinase), FAK and aurora kinase inhibitors, sensitivity to epidermal growth factor receptor (EGFR) and Janus Kinase (JAK) inhibitors in both c.del1380A CDH1 and sporadic gastric cancer cells, and relative resistance to BRD4 inhibitors in c.del1380A CDH1. Together, this study integrates transcriptomic aberrations with drug cytotoxic responses in patient-derived c.del1380A CDH1 HDGC versus CDH1 wild type sporadic gastric cancer cells providing new therapeutic leads for the difficult to treat CDH1 mutant familial subtype of gastric cancer.

## Methods

### Patient

**Fig. 1:**
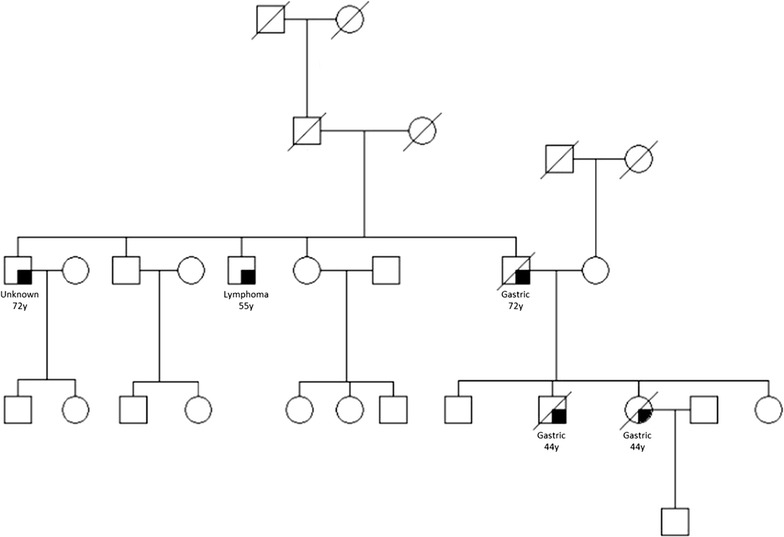
Pedigree of family with hereditary diffuse gastric cancer harboring c.1380delA germline mutation of the CDH1 gene. *Squares* indicate males; *circles* indicate females. *Symbols* with a shaded portion indicate individuals whom have been diagnosed with cancer. Type of cancer and age at diagnosis are indicated, *symbols* with *slashes* indicate deceased family members

The patient is a 44-year-old male who presented to the NIH surgical service for management of metastatic diffuse gastric cancer. All genetic counselling, clinical care, and interventions were carried out under Institutional Review Board (IRB)-approved protocol NCI-09-C-0079 with patient providing written informed consent. His sister and father were diagnosed with gastric cancer at the age of 44 and 72, respectively (Fig. 1). The patient, and other family members, previously underwent germline CDH1 mutation testing and found to harbor a heterozygous c.1380delA variant in the CDH1 locus. The patient had extensive ascites upon presentation. A palliative paracentesis was performed; cytological specimen was collected (6 L hemorrhagic ascites) and later used to establish the described patient-derived cell culture line of hereditary diffuse gastric cancer. Given the advanced stage of the patient’s cancer and his poor performance status, the patient and the NIH surgical team decided to opt for palliative care and the patient expired shortly thereafter.

### Establishment of patient-derived hereditary diffuse gastric cancer cell line

Cell suspensions created from the patient’s ascites were injected subcutaneously into female SCID mice (250,000 cells per animal). When the tumor grew to approximately 2 cm^2^, xenografts were harvested, digested, and suspended in ultra-low attachment tissue culture flasks until formation of spheroid bodies was observed [13]. Tumor-derived spheroids were then dissociated, re-seeded onto standard tissue culture flasks and grown for several passages in DMEM/F12 (Crystalgen, Commack, NY) with 10% HyClone fetal bovine serum (Thermo Scientific, Waltham, MA). Cells were passaged every 4–5 days by lifting them with 0.05% Trypsin (Gibco, Grand Island, NY), frozen after three passages, and not allowed to grow beyond 10 passaging cycles. For the spheroid assay, 1000 dissociated c.1380delA CDH1 SB.mhdgc-1 cells/mL were seeded in media conditions above in nonadherent 6-well plates coated with hydrogel (Corning Life Sciences, Chelmsford, MA) and spheroid formation observed for 6 weeks [13]. Media was replaced every 6–8 days.

### Cell lines and reagents

Establishment of tissue culture line SB.msgc-1 at our institution was previously reported [14]. Gastric cancer lines SNU-1, SNU-5, SNU-16, KATO III, AGS, NCI-N87, and BxPC-3 and HeLa cells were purchased from ATCC (American Type Culture Collection (ATCC), Manassas, VA). Antibodies for immunofluorescence studies included mouse anti-E-cadherin primary antibody (BD Transduction Laboratories, San Jose, CA), CEA/CD66e antibody (Cell Signaling, Danvers, MA), mouse IgG2a, κ (BD Biosciences, San Jose, CA), mouse (G3A1) mAb IgG1 isotype control (Cell Signaling, Danvers, MA), and Alexa Fluor ^®^ 488 goat anti-mouse IgG (H + L) antibody (Life Technologies, Frederick, MD). Etoposide, mitoxantrone, and PI-103 were purchased from SelleckChem (Houston, TX).

### Spectral karyotyping

Metaphases of SB.mhdgc-1 cells were arrested by incubation with Colcemid (KaryoMax ^®^ Colcemid Solution, Invitrogen, Carlsbad, CA) (10 μg/mL) 3 h prior to harvest. Cells were collected and treated with hypotonic solution (KCL 0.075 M) for 15 min at 37 °C and fixed with methanol: acetic acid 3:1. Slides were prepared and aged overnight for use in SKY analysis and FISH. Metaphases were hybridized with the 24-color human SKY paint kit (Applied Spectral Imaging Inc. (ASI), Carlsbad, CA) according to manufacturer’s protocol. Hybridization was carried out in a humidity chamber at 37 °C for 16 h. A post-hybridization rapid wash procedure was used with 0.4 × SSC at 72 °C for 4 min. Spectral images of the hybridized metaphases were acquired using a SD301 SpectraCubeTM system (ASI, Carlsbad, CA) mounted on top of an epi-fluorescence microscope Axioplan 2 (Zeiss, Thornwood, NY). Images were analyzed using Spectral Imaging 6.0 acquisition software (ASI, Carlsbad, CA). A minimum of 10 mitoses of comparable staining intensity and quality was examined per cell line and analyzed for chromosomal abnormality.

### Immunofluorescence microscopy

Approximately 50,000 were centrifuged onto a glass slide with Rotofix 32 A centrifuge (Hettich Lab Technology, Tuttlingen, Germany) and fixed in methanol at room temperature for 2 min. Cells were permeabilized in 0.25% TritonX-100 and blocked with 5% normal goat serum in PBS at room temperature in a humidified chamber for 2 h. Slides were incubated with mouse monoclonal anti-human E-cadherin and CEA/CD66e primary antibody, or mouse IgG2A κ or mouse G3A1 IgG1 isotype control overnight in 4 °C. Phosphoinositide signaling intermediaries were measured with anti-phosphatidylinositol 4,5-bisphosphate (PIP2) (Cat. #Z-P045) and anti-phosphatidylinositol 3,4,5-trisphosphate (PIP3) (Cat. #Z-P345; Echelon Biosciences Inc., Salt Lake City, UT) monoclonal antibodies. Alexa Fluor^®^ 488 goat anti-mouse IgG (H + L) secondary antibody was then applied for 1 h at room temperature. Slides were mounted with Vectashield/DAPI (Vector Laboratories, Burlingame, CA). Images were captured using a Zeiss LSM 510 UV (E-cadherin images) or Zeiss LSM 780 (CEA images) confocal microscope (Zeiss, Thornwood, NY).

### CDH1 mutation testing

DNA (500 ng) from both spheroid cultures and SB.mhdgc-1 monolayer cells was performed using the previously described parallel sequencing OncoVar assay [15]. In brief, Illumina paired-end adaptors were ligated to ~300 bp genomic DNA fragments. Indexing and amplification was performed using Illumina PCR primers InPE1.0 and InPE2.0 and primer indices. Pooled, indexed libraries were captured using an Agilent SureSelect Custom DNA kit targeting exons of 245 commonly mutated cancer genes (Agilent Technologies, Columbia, MD). Sequencing was done on Illumina’s Miseq sequencers. Variant calling was performed by Samtools mpileup and variants were annotated by the Annovar.

### Gene expression analysis of microarrays

**Fig. 2:**
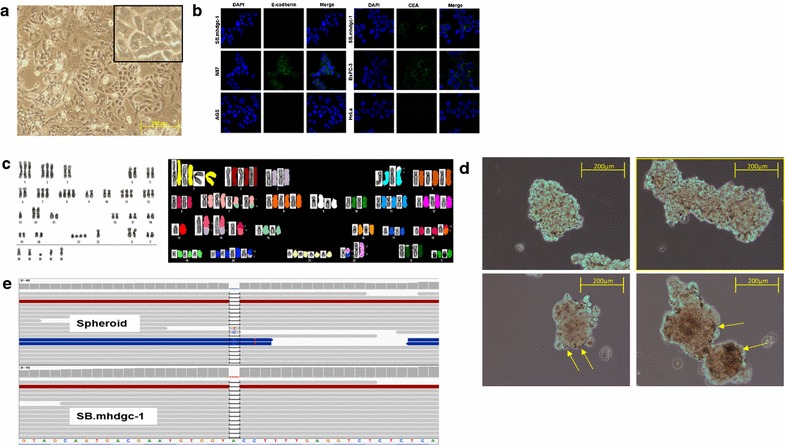
Characteristics of patient-derived c.del1380CDH1 SB.mhdgc-1 cancer cells. **a** Light microscopy image of c.del1380 CDH1 SB.mhdgc-1 cells (10× magnification). **b** SB.mhdgc-1 cells lack E-cadherin expression but express CEA. Immunoflurescence of SB.mhdgc-1 cells stained with anti-E-cadherin (*left*) and anti-CEA (*right*; both *green*), nuclei stained with DAPI (*blue*). Gastric cancer lines N87 and AGS (for E-cadherin), BxPC3 and HeLa cells (for CEA) shown as positive and negative controls. **c** Karyotype and SKY images of SB.mhdgc-1 cells show features consistent with human cancer cells including aneuploidy (such as in chromosomes 1 and 2) and chromosomal translocations (in chromosomes 3:5 and 4:8). **d** Light microscopy of c.del1380CDH1 SB.mhdgc-1 cancer cells grown under ultra-low attachment conditions in FBS-free media. *Top* (14 days of culture), three dimensional multicellular spheroid (MCS) clusters with compact, amorphous center. *Bottom* (33 days of culture), c.del1380CDH1 SB.mhdgc-1 spheroids have rounded up, became more compact, and formed basal membranes (*arrows*; 20× magnification). **e** Patient-derived SB.mhdgc-1 cells harbor c.1380del CDH1 germline mutation. Deep sequencing of Hg19 CDH1 locus (chr16: 68,771,195-68,869,444, NM_004360) in c.del1380CDH1 SB.mhdgc-1 spheroids (*top*) and SB.mhdgc-1 cells 2D monolayer cells (*bottom*); 40 base sequences of 14 genomic DNA fragments around c.1380 are shown. Artifacts of alignment as identified by BWA are shown in *blue*

Total RNA was extracted from a panel of 8 gastric cancer cell lines (SB.mhdgc-1, SB.msgc-1 and cell lines purchased from ATCC) using RNeasy Mini Kit (Qiagen, Valencia, CA). RNA concentration and integrity were analyzed with Agilent 2100 Bioanalyzer system (Agilent Technologies, Columbia, MD). Biotinylated cRNA was generated with Illumina TotalPrep™-96 RNA Amplification Kit (Life Technologies, Grand Island, NY). Biotinylated cRNA was then hybridized to HumanHT-12 v4 Expression BeadChip (Illumina, San Diego, CA); array signal was normalized to expression levels of housekeeping genes and log2-transformed. Unsupervised hierarchical clustering analysis was conducted using GeneSpring v12.6 (Agilent Technologies, Columbia, MD). Per-probe normalization was applied by subtracting the log2 signal intensity of the median value for a specific probe from the log2 signal intensity of each cell line. Genes represented by more than one probe were collapsed by aggregating to the mean. Hierarchical clustering was then performed using GeneSpring default settings, specifically Euclidean similarity measures and Wards linkage rule. Analyze Single Experiment analysis option in the default Standard Data Analysis Workflows was used for analysis. The default workflow options (Threshold: 0; *p* value: 1; Signals: both) were used for our analysis. Analyze networks (AN) algorithm with default metacore settings was used for generation of biological networks. The networks were eventually prioritized based on the number of fragments of canonical pathways. The top network is shown in Fig. 2b.

### Phospho-immunoblot analysis

Gastric cancer cells were lysed with Cell Signaling lysis buffer (Cat#40–040, Millipore, Bellerica, USA). Protein concentration was determined via BCA analysis kit (ThermoScientific, Waltham, USA). For phospho-immunoblotting using p-ERK Thr202/Tyr204 (Cat#4376), p-AKT T308 (Cat#9275), p-PDK1 Ser241 (Cat#3438) approximately 50ug of protein was loaded, for total anti-ERK (Cat#9101), AKT (Cat#C67E7), PDK1 (Cat#3062), and actin (Cat#4967, all Cell Signaling, Danvers, USA) 5–10 µg, onto 4–20% SDS/Polyacrylamide gels. Proteins were transferred to nitrocellulose blotting paper via the dry HEP-OWL1 system (ThermoScientific, Waltham, MA). Bands were visualized via the Odyssey luminescence scanner (Li-Cor, Lincoln, USA).

### Quantitative high-throughput screening (qHTS)

High-throughput drug screening was conducted in patient-derived, low passage hereditary c.del1380A SB.mhdgc-1 gastric cancer cells and low passage SB.msgc-1 derived from a metastatic lesion of a ptient with sporadic gastric cancer; the methods have been described in detail previously [16]. In brief, assays were conducted in sterile, tissue culture-treated 1536-wells. A total of 500 cells per well in 5 μL of media were seeded. Immediately after dispensing the cells, 23 nL compound solution in DMSO was transferred using a Kalypsys (San Diego, CA) pintool. Plates were then covered and incubated for 48 h. 3 μL CellTiterGlo assay reagent (Promega, Madison, WI) was added, plates were incubated for 30 min at room temperature, spun at 1000 rpm, and relative luminescence units (RLU) were quantified using a ViewLux Luminometer (PerkinElmer, Waltham, MA). The MIPE-oncology library 4.0 (MIPE: Mechanism Interrogation PlatE) contains 1912 compounds known to modulate oncology targets, pathways, and phenotypes. Additional file 1: Table S1 lists individual compounds, mechanisms of action, stage of development, structure, and acquisition information. Compounds were tested in dose–response curves starting at a final concentration 46 μM and threefold dilutions. The library was tested at 11 compound concentrations for qHTS as previously described [16, 17].

### Hit selection from qHTS and reagents list

Activity of the compounds from the dose response qHTS screen was determined based on four dose response parameters: (1) % viability at the maximum concentration of compound tested (MAXR) as a measure of compound efficacy; (2) logAC_50_ after a four-parameter fit of a complete drug response curve as a measure of compound potency; (3) curve response class (CRC) classification from dose response HTS, in which normalized data is fitted to a 4-parameter dose response curves using a custom grid-based algorithm to generate curve response class (CRC) score for each compound dose response [17, 18]. CRC values of −1.1, −1.2, −2.1, −2.2 are considered highest quality hits; CRC values of −1.3, −1.4, −2.3, −2.4 and −3 are considered inconclusive hits; and a CRC value of 4 are inactive compounds; and 4) Area Under the Curve (AUC), which is calculated using the trapezoidal rule over all measured responses [19]. (see Additional file 2: Table S2, for list of MAXR, logAC_50_, CRC, AUC, for the compounds screened in each cell line).

### Target enrichment analysis

Following selection of active compounds, we identified the annotated targets for these compounds and computed the enrichment for each target, compared to background, using Fishers exact test [20]. For this test, the background was defined as all the targets annotated in the MIPE collection. The p value from the test was adjusted for multiple hypothesis testing using the Benjamin–Hochberg method [21].

### Cell viability assay drug-response profiles

The effects of select small molecule inhibitors and chemotherapy agents identified from the qHTS on proliferation were tested by seeding 5000 cells per well in 96-well plates and incubating them for 24 h before addition of drug. Increasing concentrations of drug were added to the wells in three replicates with DMSO as negative control. Plates were analyzed 72 h after addition of drug using the Promega Cell Titer Glo assay reagent (Promega, Madison, WI). Plates were read with GloMax^®^ 96 Microplate Luminometer (Promega, Madison, WI), and the data analyzed using SoftMax version 5 and GraphPad Prism version (La Jolla, CA). Percent cell viability was calculated by normalizing raw luminescence values to vehicle-control (DMSO-treated) samples.

### Apoptosis assay

Increasing concentrations of P-103, etoposide, and mitoxantrone (all Selleck Chemicals, Houston) were added to 5000 cells seeded the day prior in 96-well plates in three replicates with DMSO as negative control. Plates were analyzed 24 h after addition of drug using Caspase-Glo^®^ 3/7 Assay System (Promega, Madison, WI). Plates were read with GloMax^®^ 96 Microplate Luminometer (Promega, Madison, WI), and the data analyzed using SoftMax version 5 and GraphPad Prism version 7 (La Jolla, CA). For cell cytometric apoptosis measurements using fragmented DNA the template-independent addition of bromolated deoxyuridine triphosphates (Br-dUTP) to free 3′-hydroxyl (OH) termini of double- and single-stranded DNA was determined using the Apo-BrdU Kit (Cat. No. 51-6536KK; BD Biosciences, San Jose, CA). 1 × 106 cells were plated, the following day DMSO, PI-103, etoposide, and mitoxantrone were added and incubated for 24 h. Cells were harvested and stained with FITC-labeled anti-BrdU monoclonal antibody according to the manufacturers instruction. Non-apoptotic (no Br-dUTP was detected) and apoptotic populations were measured on a BDFacsAriaII flow cytometer (BD Biosciences, San Jose, USA).

### Cell adhesion assay

Ratio of adherent versus floating cells at various times points after seeding was measured for SB.mhdgc-1 and SB.msgc-1 cells on regular 24-well tissues culture plates, and on 24-well plates coated with collagen I, fibronection, laminin I (all ThermoFisher Scientific, Waltham, MA), and 96-well plates coated with vitronectin (R&D Systems, Minneapolis, MN). Cells were seeded in six replicates at 1.0 × 105 cells per well in 24-well plates and 2 × 104 cells per well in 96-well plates and two independent cell counts in Nexcelom Auto T4 Hemacytometer cell chambers (Nexcelom, Lawrence, MA) were obtained. Time course ratios of adherent versus floating cells were graphed using GraphPad Prism version 7 (La Jolla, CA).

## Results

### Characteristics of hereditary diffuse gastric cancer c.del1380A CDH1 mutant SB.mhdgc-1 cells

The patient is a 44-year old male who presented for management of metastatic diffuse gastric cancer. His sister and father were diagnosed with diffuse gastric cancer at the age of 44 and 72, respectively (Fig. 1). The patient, and other family members had been previously tested for germline CDH1 mutations and found to harbor a heterozygous c.1380delA variant in exon 10 of the CDH1 locus. The protein product is a 480-amino acid long (P461Lfs*20) variant of CDH1 (Additional file 3: Figure S1), and is predicted to be disease causing and subject to nonsense-mediated RNA decay by MutationTaster and SIFT prediction leading to loss of expression. A palliative paracentesis was performed; cytological conformation of cancer cells was confirmed and used to establish the c.1380delA CDH1 SB.mhdgc-1 cancer cell line.c.1380delA CDH1 SB.mhdgc-1 grow as a pleiomorphic, irregular shaped cells monolayers to near confluency (Fig. 2a). Cells displayed considerable heterogeneity without one morphological phenotype becoming dominant after repeat passaging (>20 passages). Cells are able to raise daughter cells and cell islands both in early (<5), as well as later passages. Doubling time was prolonged between 72 and 84 h. Immunocytochemical staining shown in Fig. 2b demonstrates loss of E-cadherin expression in c.1380delA CDH1 SB.mhdgc-1 cells and strong (≥90% of cells) expression of the gastrointestinal tissue marker glycoprotein CEA. Multiple SKY karyotyping shows a diploid karyotype with frequent chromosomal aberrations including losses, duplications, and translocations typically observed in cancer (Fig. 2c). Several translocations were validated by FISH, Additional file 1: Table S1 summarizes the main detected chromosomal abnormalities. We assessed next the ability of mono-dispersed c.1380delA CDH1 SB.mhdgc-1 gastric cancer cells to self-assemble and form multicellular spheroids (MCS), both as a measure of their self-renewal ability and tumorigenicity as well as to investigate the possible impact the loss of CDH1 might have on self-aggregation and the ability to form cell-to-cell contact. Figure 2d shows different phases of MCS grown from c.1380delA CDH1 SB.mhdgc-1 cells over 6 weeks. To confirm that MCS and 2D monolayer cells maintained the same CDH1 genotype, we performed deep sequencing using the CLIA-approved OncoVar assay in both spheroids as well as monolayer c.1380delA CDH1 SB.mhdgc-1 cells confirming the unique c.1380delA CDH1 variant in both subpopulations (Fig. 2e). c.1380delA CDH1 SB.mhdgc-1 cells initiated tumors upon implantation into nude mice which failed to grow beyond 5 mm (4 months). All following experiments, including drug screening, were performed with c.1380delA CDH1 SB.mhdgc-1 cells grown as monoloyer.

The detection of the previously affirmed germline c.1380delA CDH1 germline variant in the derived cells, the loss of E-cadherin in combination with features of malignant transformation suggests the establishment of a de novo primary cell culture line from a patient with HDGC.

### Gene expression analysis of c.1380delA CDH1 mutant SB.mhdgc-1 cells shows upregulated ERK1-ERK2 and inositoltrisphosphat (IP3)/diacylglycerol (DAG) signaling in comparison to sporadic gastric cancer cells

**Fig. 3:**
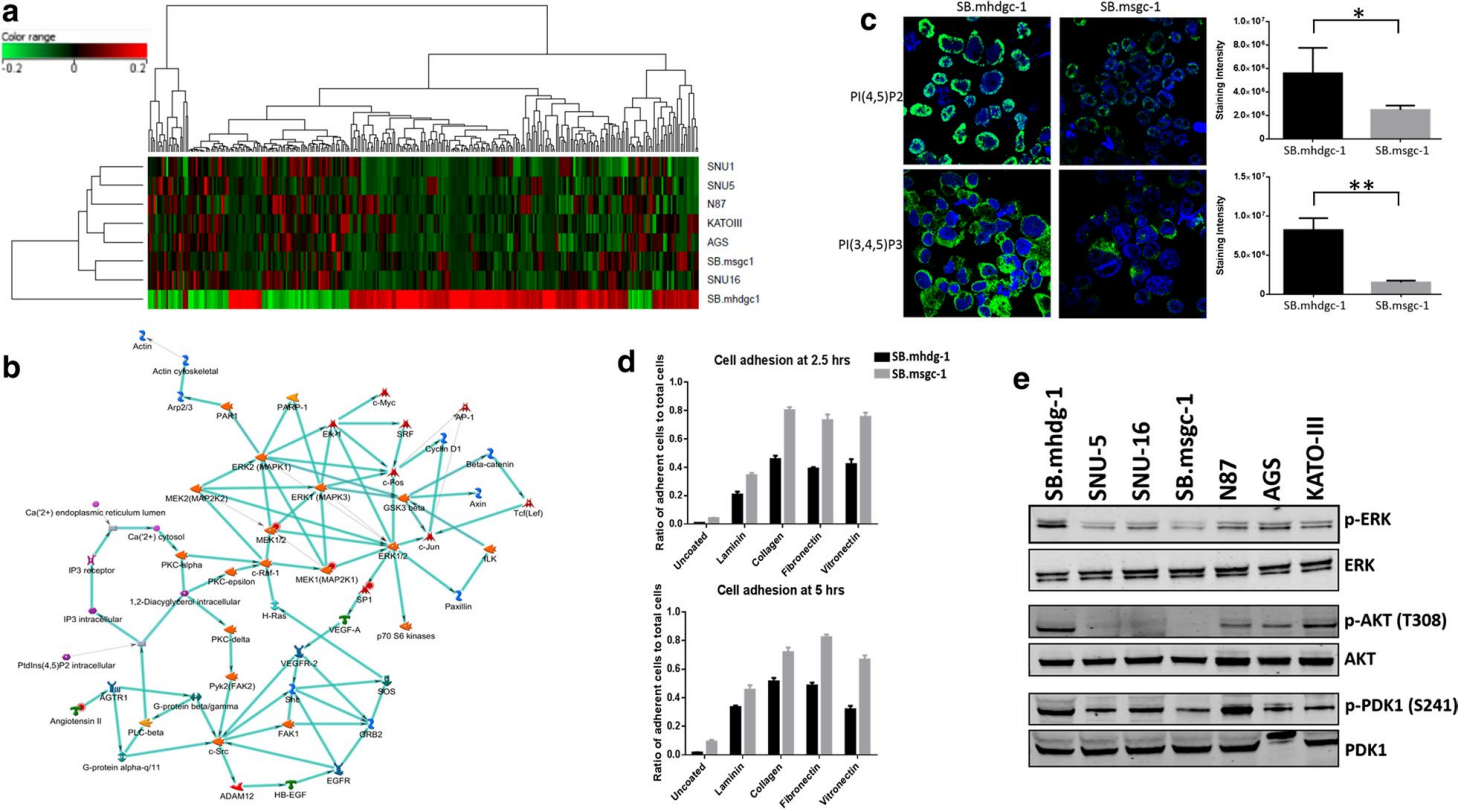
c.1380delA CDH1 SB.mhdgc-1 harbors select transcriptomic alterations compared to sporadic gastric cancer cells. **a** Unsupervised hierarchical cluster analysis and associated heat map of baseline transcriptomic profiles. *Columns* represent individual probes while rows represent individual cell lines. The color of each probe reflects log_2_ ratio of normalized expression values for each cell line compared to the median from all cell lines (see scale, top 200 upregulated and 100 downregulated (FC > 2; p < 0.05) in c.1380delA CDH1 SB.mhdgc-1 shown). **b** Most relevant network selective for SB.mhdgc-1 cells by GeneSpring GX analyze networks (AN) algorithm using shortest paths algorithm with main parameters (*1*) relative enrichment and (*2*) relative saturation of networks with canonical pathways. Networks are prioritized based on the number of fragments of canonical pathways in the network. **c** c.1380delA CDH1 SB.mhdgc-1 gastric cancer cells harbor increased phosphoinositide-derived messengers. Immunofluoresence of SB.mhdgc-1 and sporadic SB.msgc-1 gastric cancer cells measuring anti-phosphatidylinositol 4,5-bisphosphate (*top*) and anti-phosphatidylinositol 3,4,5-trisphosphate levels (*bottom*). Mean of staining intensity normalized to DAPI of 100 cells of SB.mhdgc-1 and SB.msgc-1 shown on the right. **d** Reduced cell adhesion including extracellular matrix substrate adhesion of c.1380delA CDH1 SB.mhdgc-1 versus SB.msgc-1 cells. Time course of ratios of adherent versus non-adhered cells (student’s t test; two images were acquired of each triplicate and the mean taken). **e** Increased p-ERK: total ERK and p-AKT: total AKT protein expression ratios in c.del1380A CDH1 SB.mhdgc-1 cells compared to sporadic gastric cancer cell lines. Immunoblots with antibodies indicated on the *right*

To compare the loss of CDH1 due to germline mutations in gastric cancer cells derived from a HDGC patient to sporadic gastric cancer cells, we performed genome-wide microarray profiling in c.1380delA CDH1 SB.mhdgc-1 and 7 sporadic gastric cancer cell lines using the Illumina HumanHT-12 v4 platform. Microarrays were run in triplicates, only reads passing the manufacturers QA in all replicates were included. Metastatic hereditary diffuse gastric cancer c.1380delA CDH1 SB.mhdgc-1 cells harbor distinct transcriptomic profile compared to a cohort of sporadic gastric cancer cell lines. There were 938 genes more than twofold differently regulated in the c.1380delA CDH1 SB.mhdgc-1 cells (p < 0.05; FDR < 0.1) with c.1380delA CDH1 SB.mhdgc-1 cells separating on unsupervised hierarchal cluster analysis of global gene expression profiling from sporadic gastric cancer cell lines (Fig. 3a). Gene ontology (GO) cellular process analysis of gene expression profile of c.1380delA SB.mhdgc.-1 gastric cancer cells ranked enrichment of ERK1 and ERK2 signaling first (p < 10 E-8), out of the top 10 ranked processes three involved cellular organization and cytoskeletal organization (p < 10 E-6; Additional file 4: Figure S2). To further detail signaling network perturbations selective for c.1380delA SB.mhdgc.-1 gastric cancer cells compared to sporadic gastric cancer cells, we generated biological networks using analyze networks (AN) algorithm with default settings ranking shortest paths algorithm by main parameters (1) relative enrichment with uploaded profile of c.1380delA SB.mhdgc-1 gastric cancer cells versus sporadic gastric cancer cell lines, and (2) relative saturation of networks with canonical pathways with networks are prioritized based on the number of fragments of canonical pathways on the network. Figure 3b shows the top ranked ERK1/ERK2 network (gScore 112.87; zScore 10.37; p value 1.23e-05) with MAPK pathway, inositol triphosphate (IP3)/diacylglycerol (DAG), and alternative erb receptor signaling via FAK, c-Src, or protein kinase C most prominently enriched. Other secondary networks represented in gene expression profile of c.1380delA SB.mhdgc.-1 gastric cancer cells include cytoskeletal regulators like actin, paxillin, or β-catenin.

To confirm above signal transduction aberrations observed on differential gene expression profiling of c.1380delA SB.mhdgc.-1 and sporadic gastric cancer cells, we first measured intracellular phosphatidylinositol 4,5-bisphosphate (PIP2) levels, and its metabolite phosphatidylinositol 3,4,5-trisphosphate (PIP3), as intermediaries of inositoltrisphosphat (IP3)/diacylglycerol (DAG) signaling between c.1380delA CDH1 SB.mhdgc-1 and sporadic SB.msgc-1 gastric cancer cells. SB.msgc-1 cancer cells were derived from a left liver lobe lesion of metastatic sporadic, moderately to poorly differentiated adenocarcinoma of the stomach with intestinal and signet ring cell features of a 44-years old female and has been previously described [14]. Tumor cells stained positive for Ecadherin and strongly positive (3+) for HER2 (Additional file 5: Figure S3). Levels of intracellular phosphoinositide metabolites were measured by quantitative immunofluorescence using anti-lipid phosphoinositide antibodies (Fig. 3c). Phosphatidylinositol 4,5-bisphosphate (PI(4,5)P2) is a substrate for phospholipase C–G-protein coupled receptor pathway signaling intimately involved in intracellular calcium release and overall phosphoinositide metabolism [22]. Phosphatidylinositol 3,4,5-trisphosphate (PI(3,4,5)P3)) is formed by PI 3-kinase. PI(4,5)P2 levels, and to a lesser degree, PI(3,4,5)P3 intermediaries were measured elevated in c.1380delA SB.mhdgc.-1 gastric cancer cells compared to SB.msgc-1 sporadic gastric cancer cells (Fig. 3c) commensurate with findings of comparative gene expression profiling of hereditary c.1380delA SB.mhdgc.-1 cells. To examine if detected transcriptomic perturbations in cytoskeletal regulation in hereditary c.1380delA SB.mhdgc-1 cells measured by Gene Ontology (GO) cellular process analysis and Analyze Networks (AN) algorithm might result in altered adhesion function, we compared next the ability of c.1380delA SB.mhdgc.-1 gastric cancer to SB.msgc-1 cells to adhere on regular non-treated tissue culture flasks, and then examined rescue by repeating adhesion experiments on plates coated with extracellular matrix molecules including collagen I, fibronectin, and laminin. When measuring the ratio of adherent versus non-adherent cells at 2.5 and 5 h after seeding of cells, adhesion of c.1380delA CDH1 SB.mhdgc.-1 gastric cancer cells was substantially reduced compared to CDH1 wild type sporadic SB.msgc-1 cells. The presence of extracellular matrix adhesion molecules rescued impaired adhesion of c.1380delA CDH1 SB.mhdgc-1 cells, albeit significantly less compared to SB.msgc-1 sporadic gastric cancer cells (Fig. 3d). We next measured signaling activity of the PI3K-AKT and MAPK pathway in hereditary c.1380delA CDH1 SB.mhdgc.-1 gastric cancer cells versus a panel of sporadic gastric cancer cell lines by determining ratios of phosphorylated Akt, PDK1, and ERK1/2 (MAPK42/44) to total Akt, PDK1, and ERK kinase levels. Phospho-ERK-to-total ERK, and phospho-Akt-to-total AKT ratios were elevated in c.1380delA CDH1 SB.mhdgc.-1 gastric cancer cells compared to a panel of six heterogenous sporadic gastric cancer lines (Fig. 3e), a finding in line with above gene set enrichment analysis identifying ERK1/ERK2 network as top network enriched in SB.mhdgc-1 cells. Albeit not different to sporadic gastric cancer cell lines N87 and SNU16, c.1380delA CDH1 SB.mhdgc-1 cells showed activation of the Akt-upstream regulator and PIP3 sensor 3-phosphoinositide dependent protein kinase-1 (PDK1, measured as serine 241 phosphorylation), a finding consistent with elevated PIP3 levels in SB.mhdgc-1 cells compared to SB.msgc-1 cells.

Overall, select signal transduction alterations in c.1380delA CDH1 SB.mhdgc-1 gastric cancer cells derived from differential gene expression profiling, phosphoinositide intermediary level measures, and immunoblotting suggest perturbations in MAPK kinase pathway, phosphoinositide-mediated signaling, as well as extracellular matrix adhesion dysfunction enriched in c.1380delA CDH1 SB.mhdgc-1 gastric cancer cells compared to sporadic gastric cancer cell lines.

### Dose response quantitative high-throughput screening of c.1380delA CDH1 SB.mhdgc-1 and sporadic gastric cancer cells SB.msgc-1 with an oncology library identifies selective pharmacological vulnerabilities in hereditary diffuse gastric cancer cells

**Fig. 4:**
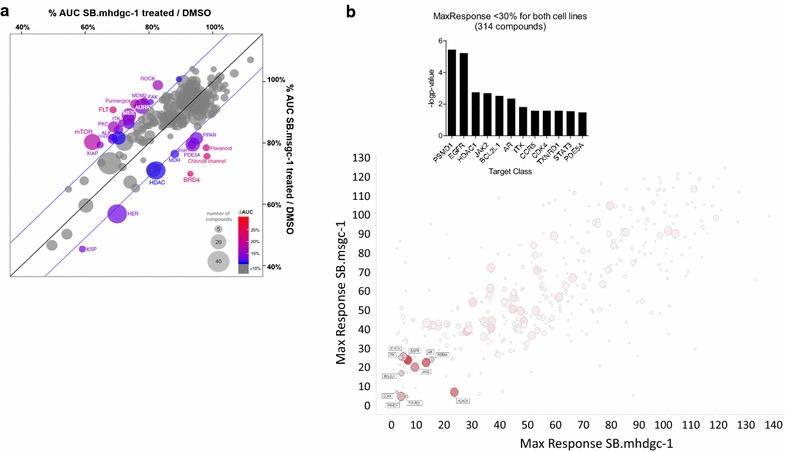
Comparative quantitative high-throughput screening (qHTS) using compounds from the MIPE Oncology 4.0 library identifies compounds with preferential activity against c.del1380A CDH1 SB.mhdgc-1 cells compared to SB.msgc-1 gastric cancer cells. **a** Bubble diagram of drug phenotypes by compound class of SB.mhdgc-1 versus SB.msgc-1 cells depicting class activities (number of compounds per drug class) measured by area under the curve (AUC) (drug class activities ≥2 standard deviations from the mean of ΔAUC(% AUC(c.1380delA SB.mhdgc-1/SB.msgc-1) being considered significantly more active in the respective cell line). **b** Drug classes with activity in both c.del1380A CDH1 SB.mhdgc-1 and SB.msgc-1 gastric cancer cells by maximum response (MAXR) <30%. Enrichment (number of active compounds in a target class relative to total number of compounds for that target class versus enrichment for each target, compared to background; Fishers exact test, 2-tailed)

Adherent c.1380delA CDH1 SB.mhdgc-1 cells, derived from a patient with hereditary diffuse gastric cancer due a germline CDH1 mutation, and adherent SB.msgc-1 gastric cancer cells, derived from a patient with sporadic gastric cancer, were screened in 1536-well microplates for growth inhibition using the MIPE 4.0 Oncology Library which includes 1912 oncology compounds which are either clinically approved or currently in late preclinical development. Full compound dose response curves starting from 46 µM and threefold dilutions were used to treat the cells for 48 h. Drug effects were quantitated by four different methods capturing efficacy (MAXR, maximum response), potency (LogAC_50_), or both, CRC scores (Additional file 6: Figure S4), and area under the curve (AUC). The results of the dose response screening of the MIPE 4.0 oncology collection show that each cell line had distinct pharmacological vulnerabilities regardless of the dose response curve parameter used to determine compound activity. Area under the curve (AUC) is a parameter often used to quantitate the effect of drugs because it integrates both efficacy and potency. Figure 4 shows a bubble chart, correlation plot depicting both the number of compounds per drug class, as well as the overall AUC activity of the compounds within a target class for both cell lines. Drug class activities with ≥2 standard deviations from the mean of ΔAUC(% AUC(c.1380delA SB.mhdgc-1/SB.msgc-1) were considered significantly more active in the respective cell line. Using ΔAUC, drug classes with enriched activity against hereditary diffuse gastric cancer c.1380delA CDH1 SB.mhdgc-1 cells include mTOR, MEK, FAK, ROCK, and protein kinase C inhibitors, with aurora kinase inhibitors showing a trend towards greater activity, whereas bromodomain-containing protein 4 (BRD4), phospho-diesterase (PDE5A) inhibitors, and to a lesser degree, HDAC and HER receptor inhibitors showed preferentially greater activity in the sporadic wild type CDH1 SB.msgc-1 cells. Using the MAXR (cell killing at highest tested drug concentrations) parameter as a measure of compound activities, the drug profiles and drug classes measured as active in the two cell lines were similar to those obtained using AUC (Additional file 7: Figure S5A). Using potency (logAC_50_) as a measure of compound activity, fewer drug classes were significantly different between c.1380delA CDH1 SB.mhdgc-1 and SB.msgc-1 cells: while topoisomerase II and AKT inhibiting agents were identified as preferentially active, some of the previously differently observed drug classes like MEK, protein kinase C, or ALK inhibitors were not measured as different activity profiles in the two cell lines (Additional file 7: Figure S5B) when using logAC_50_. When applying the most stringent criteria for drug activity by combining both criteria high quality drug response curve (drugs yielding high quality CRCs including −1.1, −1.2, −2.1, and −2.2) and potency (Δ-log[AC50] (SB.mhdgc-1–SB.msgc-1) < 1) and comparing drug activity profiles between c.1380delA CDH1 SB.mhdgc-1 and SB.msgc-1 cells, compounds targeting mTOR, PI3KCA, and AKT1 were significantly (p value < 0.01) enriched as selectively active in hereditary diffuse gastric cancer versus sporadic SB.msgc-1 cells (Additional file 8: Figure S6A). Box plots showing the logAC50 distribution of logAC50 for the compounds of targets showing enrichment as being more selectively potent for c.1380delA CDH1 SB.mhdgc-1 shown in Additional file 8: Figure S6B identifies activities of anti-mTOR, topoisomerase II, and tubulin inhibitors as most different between the two cell lines. Since dysregulated gene expression of druggable targets can indicate dependency, and possibly pharmacological vulnerability in affected cells, we overlaid differential drug phenotype and gene expression changes (fold difference) between c.1380delA CDH1 SB.mhdgc-1 and SB.msgc-1 cells of known direct druggable targets of any of the compounds represented in the MIPE 4.0 Oncology Library. Only 52 of the direct targets of compounds in the MIPE collection showed greater than twofold difference in expression levels between c.1380delA CDH1 SB.mhdgc-1 and SB.msgc-1 cells. Using difference in potency (delta logAC_50_) and maximum response percent viability upon treatment of both cell lines shown as overlaid boxplots in Additional file 8: Figure S6C, the most distinct correlations between dysregulated genes and pharmacological profile of c.1380delA CDH1 SB.mhdgc-1 versus SB.msgc-1 cells included anti-topoisomerase II and anti-microtubule (TUBB) regulation. As the above differential screening approach missed common pharmacological vulnerabilities shared between c.1380delA CDH1 SB.mhdgc-1 and SB.msgc-1 cells, which may harbor valuable future therapeutic leads, we also examined activities screen results for active hits present in both cell lines using MAXR. There were a total of 314 compounds which ≥70% complete cell killing at highest drug concentration (Fig. 4b). Classes with significant enrichment (number of active compounds in a target class relative to the total number of compounds for that target class; computed enrichment for each target, compared to background, using Fishers exact test; p < 0.01) included anti-EGFR and JAK2 inhibitors (Fig. 4b).

In summary, hereditary c.1380delA CDH1 SB.mhdgc-1 have select pharmacological vulnerabilities which, in part, align with signal transduction perturbations and gene expression alterations detected in these cells.

### Validation of candidate active compounds in c.1380delA CDH1 SB.mhdgc-1 cells

**Fig. 5:**
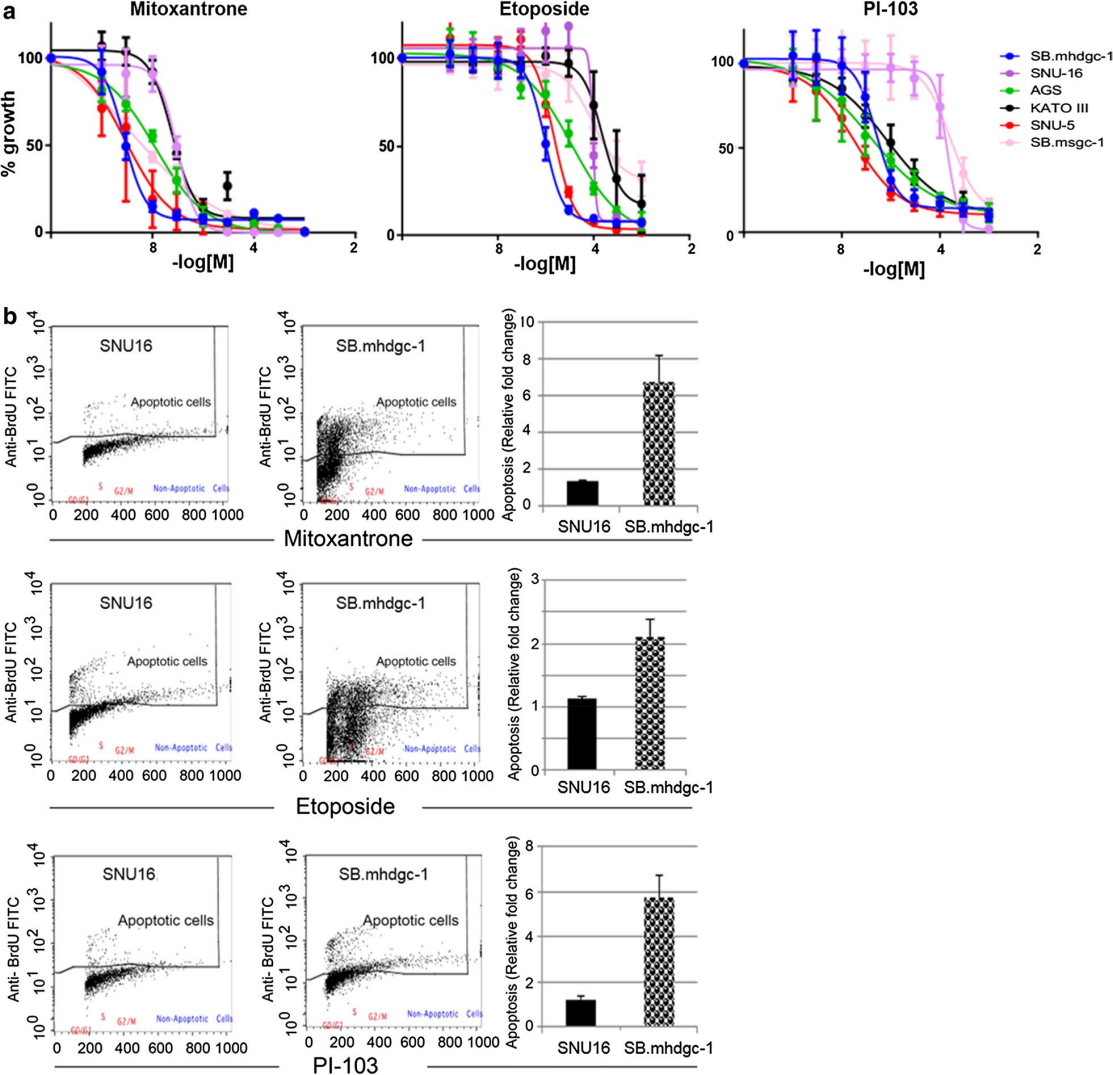
c.1380 CDH1 SB.mhdgc-1 gastric cancer cells show vulnerabilities to toposisomerase II and PI3K/mTOR inhibition. **a** Drug response curves of a panel of gastric cancer cell lines treated with a range of concentrations of mitoxantrone, etoposide (both TOPO2A inhibitors), or PI-103 (dual class IA phosphatidylinositol 3 kinase/mTOR inhibitor) for 72 h. *X-axis* indicates log [concentration] tested, *y-axis* indicates cell viability percentage normalized to vehicle-control samples. Mean cell viability values are plotted with standard error of the mean (SEM) from at least 2 independent experiments done in triplicate. **b** Rate of apoptosis induced by 24-h treatment of 1 µM etoposide, mitoxantrone, or PI-103 normalized to DMSO-treated samples in sporadic gastric cancer SNU-16 and hereditary c.1380delA SB.mhdgc-1 cells. Flow cytometry profiles of FITC-labeled anti-BrdU staining of 3′-hydroxyl (OH) termini of double- and single-stranded DNA, relative BrdU fractions normalized to DMSO-treated control shown on the *right*

Based on both the enrichment profile of preferentially active drugs as well as gene expression and signal transduction aberrations in c.1380delA CDH1 SB.mhdgc-1 gastric cancer cells, we selected the dual PI3K/mTOR inhibitor PI-103 and the topoisomerase II inhibitors etoposide and mitoxantrone as leads for further follow-up. One of the other candidates targeting microtubule assembly and disassembly, anti-tuberin agent taxane, is already used in the management of advanced gastric cancer, including in hereditary diffuse gastric cancer, and we elected not to extend studies on this class of agents further. We first aimed to validate the increased activity of the dual PI3K/mTOR and topoisomerase II inhibitors in hereditary c.1380delA CDH1 SB.mhdgc-1 gastric cancer cells in an extended panel of sporadic gastric cancer cell lines. Figure 5a shows full 10-point dose response curves of PI-103, etoposide, and mitoxantrone confirming select sensitivity of hereditary c.1380delA CDH1 mutant SB.mhdgc-1 gastric cancer cells compared to most sporadic gastric cancer cells (up to 100- to 1000-fold lower GI50 measurements compared to SNU-16 or SB.msgc-1 cells). Next, we examined apoptosis levels upon treatment in c.1380delA CDH1 SB.mhdgc-1 and sporadic gastric cancer lines. Levels were initially measured via a caspase-3 and 7 chemoluminescence screening assay at 1 µmol drug treatment which showed elevated levels of activated caspase-3 and -7 in c.1380delA CDH1 SB.mhdgc-1 cells (Additional file 9: Figure S7). Flow cytometry analysis of cell death measured by labeling DNA breaks with FITC-dUTP confirmed increased fraction of BrU positive cells in mitoxantrone, etoposide, and PI-103-treated hereditary c.1380delA CDH1 mutant SB.mhdgc-1 gastric cancer cells showed increased cell death compared to sporadic gastric cancer cells (Fig. 5b). These findings, in line with results of above qHTS screen result, may hence provide possible leads for future preclinical, and clinical, studies.

## Discussion

Recent large-scale molecular subtyping efforts have opened novel individualized treatment avenues for gastric cancer [23–25]. For example, patients with metabolic subtype of gastric cancer derived from large gene expression profiling of multiple cohorts of clinical gastric cancer specimens show improved clinic outcome upon treatment with 5-FU [23]. While cancer cells of the metabolic subtype were measured more sensitive to 5-FU, gastric cancer cells of the mesenchymal subtype responded to a greater degree to PI3K/mTOR inhibition [23]. Similarly, the recently released data from The Cancer Genome Atlas (TCGA) initiative suggests genomically defined leads, for example, of increased sensitivity to receptor tyrosine kinase or cell cycle modulating agents in the chromosomal unstable (CIN) identified gastric cancers, or increased sensitivity to PI3K kinase, JAK2, and immune checkpoint blockade in the EBV positive subtype [25]. In contrast, leads for drug sensitivities unique to familial gastric cancer due to germline CDH1 mutations are to date largely derived from a synthetic lethality and drug screen in an isogenic CDH1 knockout breast fibroblast model (MCF10A CDH1(−/−)) [12], CDH1-negative systems overexpressing wild type versus mutant forms of CDH1 [26, 27], or correlative tissue studies of the early unique T1a lesions of prophylactic gastrectomy specimens [8, 10]. That hereditary diffuse gastric cancer (HDGC) due to germline CDH1 variants, and sporadic gastric cancers with somatic CDH1 perturbations are unique subtypes of gastric cancer, likely to harbor different drug sensitivity profiles and hence offer opportunities for genotype-directed personalized treatment approaches, is supported by a number of observations; for example, in a detailed CDH1 profiling effort of 174 sporadic and 72 familial gastric cancer specimens Corso et al. reported significant differences in clinical outcomes depending on presence and type of CDH1 alternation with the worst survival rates observed across all examined gastric cancers in cases with structural CDH1 defects and in particular in familial tumors [11]. Or, eleven percent of the 205 primary gastric adenocarcinoma, examined as part of the TCGA effort, harbored somatic mutations in the CDH1 gene, which occurred nearly exclusively in the genomically stable (GS) gastric cancer subtype (37% of GS cases) which, by far, comprised of the largest number of cancers with diffuse histology [25].

In order to identify treatment leads selective for the HDGC subtype due to CDH1 germline mutations of gastric cancer, we derived a primary tissue culture line from an HDGC patient with a c.1380delA CDH1 truncating germline mutation leading to loss of CDH1 expression, and compared the transcriptomic profile and drug sensitivity profile of these cells to a panel of sporadic gastric cancer cells. GO analysis identified upregulation of ERK and phosphoinositide-mediated signaling network processes in hereditary c.1380delA CDH1 SB.mhdgc-1 gastric cancer cells which showed selective sensitivities to different drug classes including mTOR, MEK as well as c-Src, FAK, or topoisomerase II inhibiting agents. These findings of select drug response profiles of hereditary SB.mhdgc-1 gastric cancer cells to MEK and mTOR inhibition, in combination with gene expression dysregulation of ERK and phosphoinositide-mediated signaling networks, also appear to be in line with elevated phospho-ERK: total ERK and phospho-Akt (T308): total Akt ratios observed as a measure of dysregulated signal transduction activity in SB.mhdgc-1 cells. Additionally, these results, in part, overlap with data obtained from comparative gene expression profiling, synthetic RNA lethality and drug screening in the isogenic MCF10A CDH1(−/−) breast fibroblast model as well as prior large-scale gene profiling studies of sporadic clinical gastric cancer specimens [12, 23, 28].

Similar to the MCF10A CDH1(−/−) mutant cells, c.1380delA CDH1 SB.mhdgc-1 gastric cancer cells showed altered gene expression of genes involved in cellular component organization, cytoskeletal organization, and cell adhesion (Additional file 4: Figure S2), including microtubule nucleation involving genes like TUBB2 [28]. We found c.1380delA CDH1 SB.mhdgc-1 cells sensitive to taxanes targeting TUBB1 as well as agents targeting mitosis like the aurora kinase inhibitors, a finding also made by Telford and colleagues in the MCF10A CDH1 (−/−) system [12, 29]. Elevated phosphoinositide signaling, both by direct detection of PI(4,5)P2 and PI(3,4,5)P3s messenger intermediates as well as by network analysis of differential gene expression profiling may be a consequence of increased GPCR signaling, which was the most enriched functional cluster (enrichment score = 10.01) in the synthetic lethality screen and confirmed by increased sensitivity to JAK2 inhibition, an immediate downstream effector kinase of GPCR signaling complexes, in the isogenic MCF10A CDH1 (−/−) system studied by Telford et al. [12]. Overall, drug sensitivities with overlap to Telford and colleagues’ findings in the CDH1(−/−) isogenic mutant MCF10A model include inhibitors of the PI3K/mTOR axis, including PI-103 followed up in our validation studies, mTOR, aurora kinase inhibitors as well as inhibitors of c-Src kinase. Our findings are also in line with the characteristics, both on a molecular and drug sensitivity level, of a large gene expression profiling effort across 258 tumors which identified three subclasses of gastric cancer [23]. In this large study of well-validated patient specimens, gene expression analysis identified three subgroups, mesenchymal, proliferative and metabolic, based on transcriptomic differences. The mesenchymal subtype most frequently harbored diffuse gastric cancers (up to 92.5%), showed cell adhesion and cell motility as well as focal adhesion and ECM receptor gene expression aberrations and displayed sensitivity to inhibitors of the PI3K/AKT pathway. These tumors were frequently hyper-methylated and of high grade and appear to be most similar to the genomically stable (GS) subtype by TCGA which consists of 75% Lauren classification type diffuse tumors, which has the highest rate of CDH1 mutations, as well as frequent variants involving genes of cytoskeletal, cell polarity, and cellular component organization, both findings in line with the GO process analysis and drug phenotype in the c.1380delA CDH1 SB.mhdgc-1 cells [23, 25]. On the other, there were also significant differences between the pharmacological profile of MCF10A CDH1 (−/−) mutant cells reported by Telford and coworkers and the drug phenotype of c.1380delA CDH1 SB.mhdgc-1 in our study. Among these, for example, sensitivity to MEK or EGFR inhibition was not seen in E-cadherin deficient MCF10A cells and sensitivity to the c-Src kinase inhibitor saracatinib was only shown at one of the three concentrations tested to be significant between the CDH1 MCF10A isogenic cell line pair with overall generally a more modest effect on cell viability [12]. Also, there was discordancy between the sensitivities to HDAC inhibitors and anti-apoptosis inhibitors to BCL2 and XIAP between the two systems. We attribute these differences to the more evolved stage of the patient-derived c.1380delA CDH1 SB.mhdgc-1 cells compared to the MCF10A system. In the sentinel studies of Humar and colleagues, who examined signal transduction aberrations of the T1a stage with phospho-immunohistochemistry in a family with a c.1008G>T CDH1 germline mutation, detailed pathology analysis showed that deficiency in E-cadherin is sufficient to initiate diffuse gastric cancer in the absence of hyperproliferation and that early intramucosal signet-ring cell carcinoma (SRRC) is initially slow proliferating in the upper neck of the gastric glands with loss of expression of junctional molecules including actin, p120, or Lin-7 homologue A of the cell polarity complex [9, 10]. Expansion and progression beyond the early HDGC base is associated with c-Src kinase activation, including activation of downstream effectors FAK and signal transducer and activator of transcription 3 (STAT3), and described as one of the sentinel events of progression beyond the gastric mucosa and transformation to poorly differentiated cells and the development of an EMT phenotype [10]. It is thus perceivable that the MCF10A system, which is not tumorigenic per se and has no oncogenic addiction, represents the very early intramucosal T1a stage in the slow proliferating phase with no, or limited, response to inhibitors c-Src inhibitors but capturing the perturbations associated with defective CDH1 adhesion and cell polarity signaling, whereas the transformed c.1380delA CDH1 SB.mhdgc-1 cells derived from the ascites of the HDGC patient with diffuse gastric cancer are dependent on c-Src, MAPK kinase, or other late occurring signal transduction signaling aberrations [12, 28]. It is known that elevated c-Src or MAPK signaling pathways are involved in the activation of EMT transcription factors [30–32]. Thus, while previous results including pharmacological leads from the isogenic E-cadherin deficient MCF10A model, or from a study in a CDH1 null drosophila model, might be mostly applicable to the early, intramucosal T1a stage, we propose that leads derived from patient-derived c.1380delA CDH1 SB.mhdgc-1 cells capture later tumor stages shifting from a drug profile with primarily chemopreventative merit to a therapeutic one. The anecdotal use of anti-EGFR therapy with cetuximab in HDGC patients with advanced gastric cancers applied by some medical oncologists in the field appears to be in line with the detected sensitivities to EGFR, PI3K and MEK inhibition as well as elevation of MAPK kinase signaling in c.1380delA CDH1 SB.mhdgc-1 cells. Similar to KRAS wild type colon cancer, in the absence of constitutively active RAS reduction of upstream input to the MAPK and PI3 kinase signal transduction pathways, EGFR inhibition might have merit as a molecular therapy option which might be improved by the use of select downstream mTOR inhibition considering alternative non-erb receptor ligand activation of the PI3K pathway via increased phosphoinositol-mediated signaling, c-Src or protein kinase C signaling via ERK-mediated release of TSC1/2 and mTORC1 inhibition.

Together, combined comparative gene expression profiling and qHTS in patient-derived hereditary c.1380delA CDH1 SB.mhdgc-1 cells may open new avenues to improved individualized treatment options for familial gastric cancer.

## Conclusion

Hereditary diffuse gastric cancer due to CDH1 germline mutations has to date largely escaped the benefits of the personalized medicine approach. While significant progress with regard to improvements in screening, surveillance, and risk reducing interventions has been made, HDGC patients affected by advanced gastric cancer have few effective treatment options, and receive, in large, despite the significant clinicopathological and genetic differences, the same systemic treatment options like patients affected by sporadic gastric cancer. Using the patient-derived c.1380delA CDH1 SB.mhdgc-1 and SB.msgc-1 tissue culture lines, and an extended panel of sporadic gastric cancer cell lines, combined comparative gene expression profiling and qHTS drug screening with a large oncology library identified leads with selective activity in familial gastric cancer cells occurring in the context of CDH1 germline mutations. Some of the validated leads have regulatory approval for other oncology indications, and thus can be expeditiously translated into early clinical trial testing possibly opening new avenues to improved treatment options for patients with familial gastric cancer.

## Additional files

**Additional file 1: Table S1.** Chromosomal aberrations detected by FISH in SB.mhdgc-1.

**Additional file 2: Table S2.** Quantitative high-through put drug screening using MIPE Oncology 4.0 library in hereditary c.delA1380 CDH1 SB.mhdgc-1 and sporadic SB.msgc-1 cells.

**Additional file 3: Figure S1.** Protein product of c.1380delA CDH1 variant predicted by MutationTaster. Wild type CDH1 sequence (UniProt ENST00000261769) shown on top, c.1380delA CDH1 variant on bottom.

**Additional file 4: Figure S2.** Top ten Gene Ontology (GO) cellular processes differently regulated in c.1380delA CDH1 SB.mhdgc-1 versus panel of sporadic gastric cancer cells. Enrichment analysis of all genes with FC > 2; p < 0.05 and q < 0.05 was carried out using GeneGo Metacore data mining and analysis software (online version; http://portal.genego.com). Probabilities of a random intersection between a set of IDs the size of target list with ontology entities estimated in p value of hypergeometric intersection (top; the lower p value means higher relevance of the entity to the dataset, which shows in higher ranking for the entity).

**Additional file 5: Figure S3.** E-cadherin (left) and HER2 expression in metastatic, moderately to poorly differentiated adenocarcinoma of the stomach SB.msgc-1. Immunohistochemical staining at magnification ×20, inlet ×40.

**Additional file 6: Figure S4.** Diagrams of different dose responses and the corresponding curve response class (CRC) scores. CRC parameters integrate potency and efficacy measurements of the compounds. Drug response curves with CRC −1.1 exhibit a near complete maximum response; −1.2 exhibit less effective maximum cell killing; −2.1 do not have a maximum response but can achieve killing of nearly all the cells; −2.2 do not have a maximum response and can only achieve intermediate killing; and 4 are inactive.

**Additional file 7: Figure S5.** Selective pharmacological vulnerabilities of c.1380delA SB.mhdgc-1 versus SB.msgc-1 gastric cancer cells identified by comparative qHTS screening with the MIPE Oncology 4.0 library. **A**, Bubble diagram of drug phenotypes by compound class of SB.mhdgc-1 versus SB.msgc-1 cells depicting class activities (number of compounds per drug class) measured by maximum response (max response SB.mhdgc-1 / max response SB.msgc-1). **B**, Bubble diagram comparing drug activities measured by potency (logAC_50_SB.mhdgc-1 versus logAC_50_SB.msgc-1).

**Additional file 8: Figure S6.** **A**, Target enrichment for compounds with CRC −1.1, −1.2, −2.1, or −2.2 and delta logAC_50_ (SB.mhdgc-1 logAC_50_–SB.msgc-1 logAC_50_) < −1. −log p values were calculated as described in materials and methods based on the total number of compounds targeting a gene or mechanism. **B**, LogAC_50_ distributions of compounds that show CRC −1.1, −1.2, −2.1, or −2.1 and logAC50 <−1 organized by enriched target class in c.1380delA CDH1 SB.mhdgc-1 versus SB.msgc-1 gastric cancer cells. Box plots of median logAC_50_ values of select target classes showing enrichment in c.1380delA CDH1 SB.mhdgc-1 (red plots) and SB.msgc-1 (blue plots). **C**, Concomitant dysregulation of target genes and selective activity of MIPE compounds in c.1380delA CDH1 SB.mhdgc-1 versus SB.msgc-1 gastric cancer cells. Genes with greater than two-fold expression difference in c.1380delA CDH1 SB.mhdgc-1 compared to SB.msgc-1 cells and which are direct targets of compounds in MIPE Oncology 4 are shown. Genes that are downregulated in c.1380delA CDH1 SB.mhdgc-1 compared to SB.msgc-1 cells are represented by green dots; red dots represent upregulated genes (log10 fold change indicated on the right). Overlaid are boxplots for difference in logAC_50_ (LAC_50_) SB.mhdgc-1–SB.msgc-1 for all compounds in MIPE Oncology 4 per target (top) or maximum response (bottom).

**Additional file 9: Figure S7.** Increased induced cell death upon treatment with topoisomerase II and dual PI3K/mTOR in hereditary c.1380delA CDH1 SB.mhdgc-1 versus sporadic gastric cancer cell lines. Caspase 3/7 levels after 24 h of treatment with 1 µM mitoxantrone, 1 µM etoposide, or 1 µM PI-103 normalized to DMSO-treated control is shown (standard errors of the mean from at least 2 independent experiments done in triplicate shown).

## Abbreviations

ALK: Anaplastic Lymphoma receptor tyrosine Kinase
CDH1: E-cadherin
DAG: diacylglycerol
EGFR: epidermal growth factor receptor
EMT: epithelial–mesenchymal transition
ERK: extracellular signal regulated kinase
FAK: focal adhesion kinase
HDGC: hereditary diffuse gastric cancer
IP3: inositol trisphosphate
JAK: Janus Kinase
MEK: mitogen-activated protein kinase
mTOR: mammalian target of rapamycin
PI3K: phosphatidylinositol 3-kinase
PKC: protein kinase C
STAT3: signal transducer and activator of transcription 3
TOPO2: topoisomerase II
