# Supplementary material for: Transcriptomic profiling and quantitative high-throughput (qHTS) drug screening of CDH1 deficient hereditary diffuse gastric cancer (HDGC) cells identify treatment leads for familial gastric cancer
Source: J Transl Med. 2017 May 1;15:92. doi: 10.1186/s12967-017-1197-5 (PMC5412046; doi:10.1186/s12967-017-1197-5)
Supplement: Supplementary file 1 — Additional file 1: Table S1. Chromosomal aberrations detected by FISH in SB.mhdgc-1. [file 12967_2017_1197_MOESM1_ESM.docx]

**Table S1**. Chromosomal aberrations detected by FISH in SB.mhdgc-1.

| **Translocations** | **Insertions** | **Deletions** |
| --- | --- | --- |
| t(3;5) | i(12p) | del(5) |
| t(4;8) | i(12q) | del(8) |
| t(6;8) |  |  |
| t(6;12) |  |  |
| t(17;9) |  |  |
| t(10;13) |  |  |
| t(18;Y) |  |  |
| t(X;Y) |  |  |
