# Supplementary figures and images for: Transcriptomic profiling and quantitative high-throughput (qHTS) drug screening of CDH1 deficient hereditary diffuse gastric cancer (HDGC) cells identify treatment leads for familial gastric cancer
Source: J Transl Med. 2017 May 1;15:92. doi: 10.1186/s12967-017-1197-5 (PMC5412046; doi:10.1186/s12967-017-1197-5)

## Slide 1
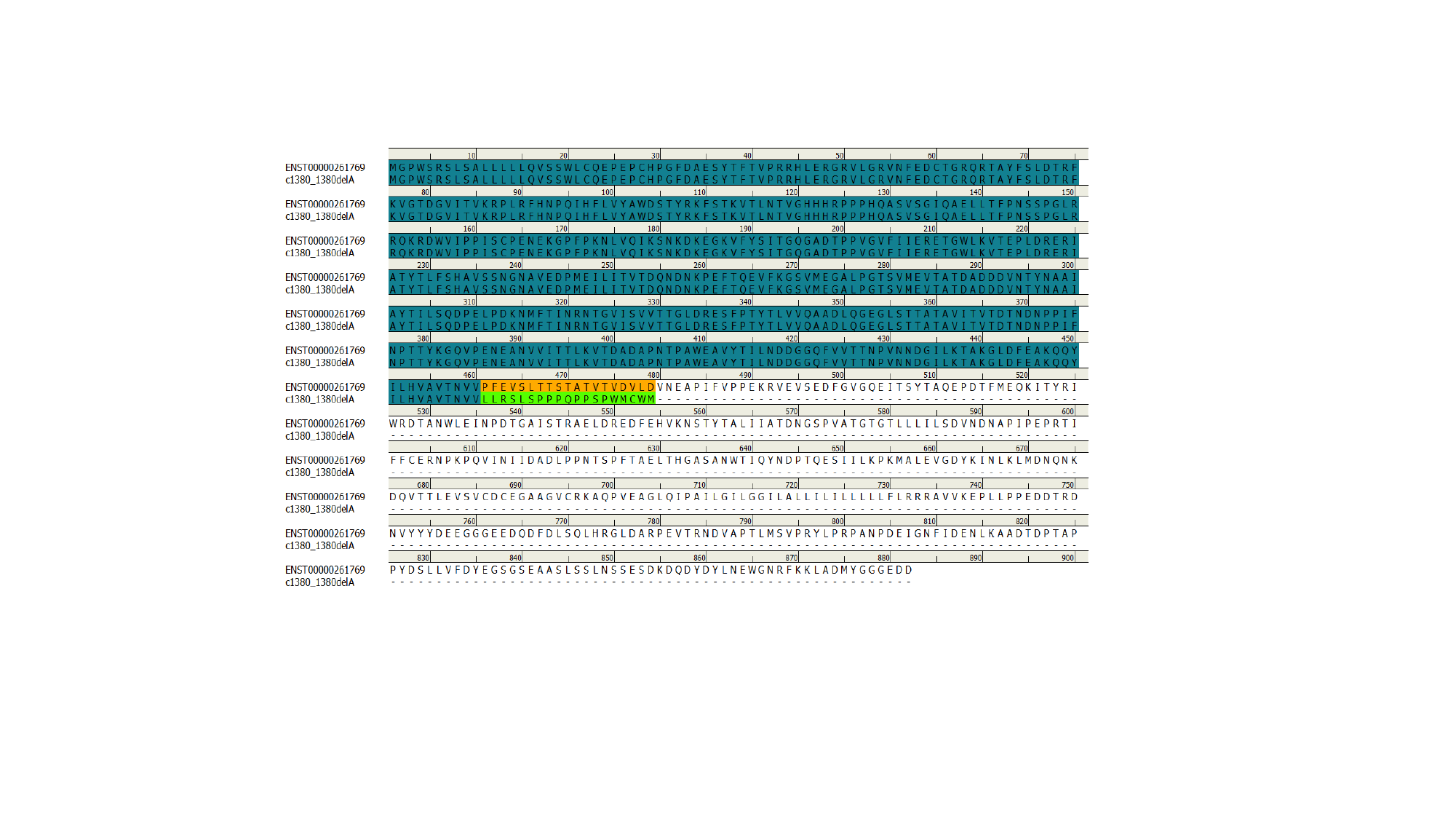

Supplement: Supplementary file 3 — Additional file 3: Figure S1. Protein product of c.1380delA CDH1 variant predicted by MutationTaster. Wild type CDH1 sequence (UniProt ENST00000261769) shown on top, c.1380delA CDH1 variant on bottom. [file 12967_2017_1197_MOESM3_ESM.pptx]

## Slide 1
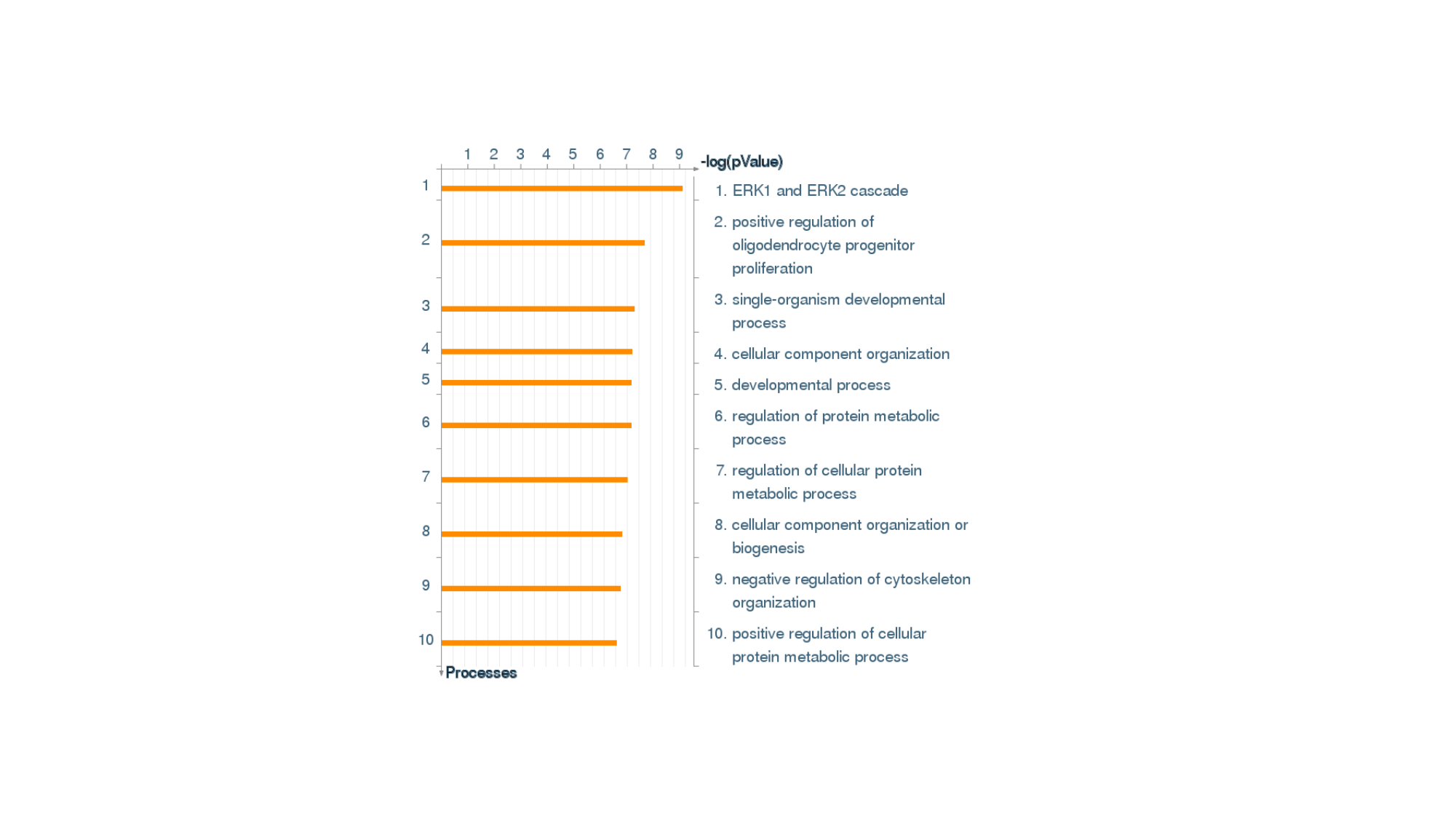

Supplement: Supplementary file 4 — Additional file 4: Figure S2. Top ten Gene Ontology (GO) cellular processes differently regulated in c.1380delA CDH1 SB.mhdgc-1 versus panel of sporadic gastric cancer cells. Enrichment analysis of all genes with FC > 2; p < 0.05 and q < 0.05 was carried out using GeneGo Metacore data mining and analysis software (online version; http://portal.genego.com). Probabilities of a random intersection between a set of IDs the size of target list with ontology entities estimated in p value of hypergeometric intersection (top; the lower p value means higher relevance of the entity to the dataset, which shows in higher ranking for the entity). [file 12967_2017_1197_MOESM4_ESM.pptx]

## Slide 1
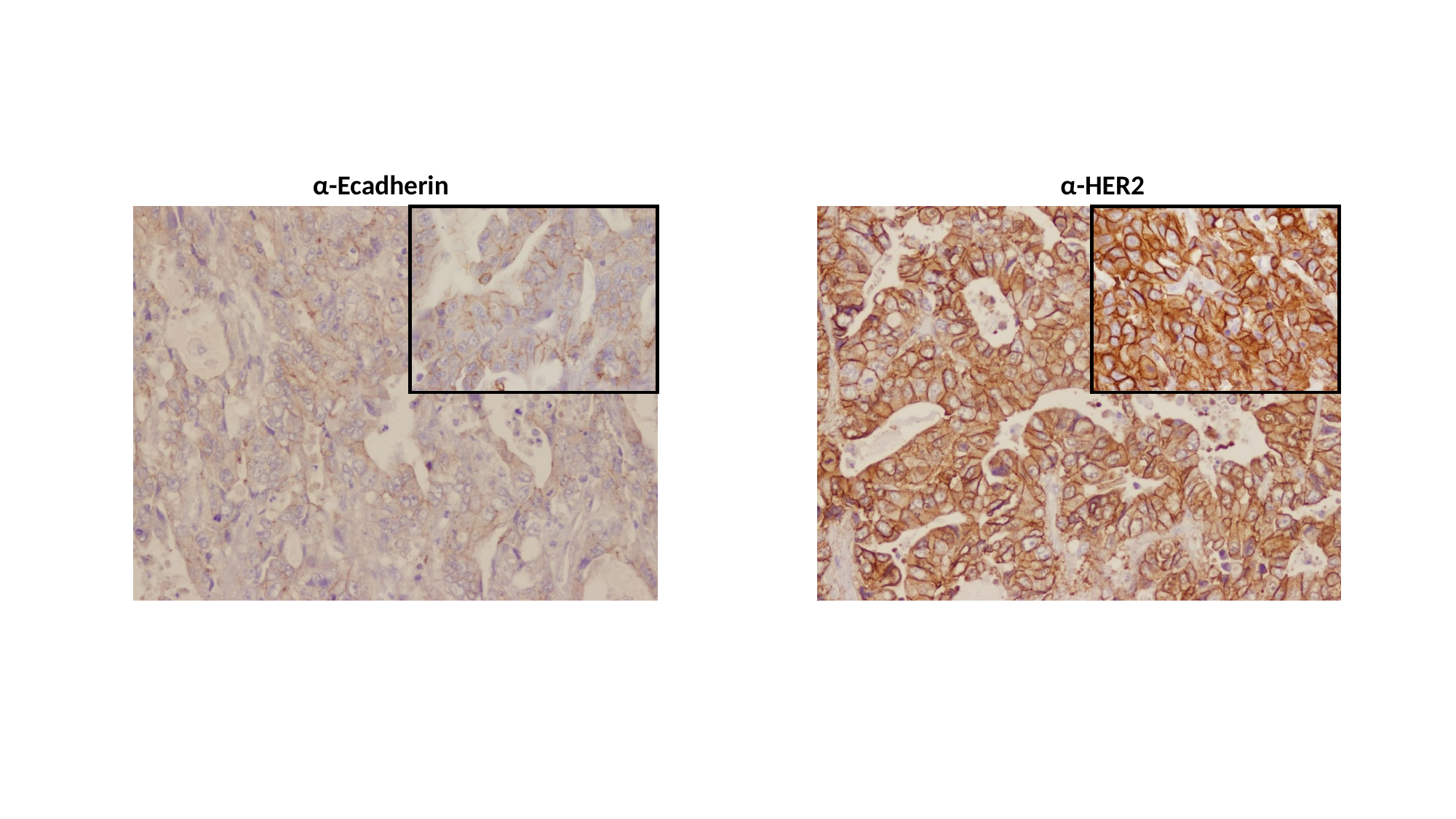

α-Ecadherin				 	 α-HER2

Supplement: Supplementary file 5 — Additional file 5: Figure S3. E-cadherin (left) and HER2 expression in metastatic, moderately to poorly differentiated adenocarcinoma of the stomach SB.msgc-1. Immunohistochemical staining at magnification ×20, inlet ×40. [file 12967_2017_1197_MOESM5_ESM.pptx]

## Slide 1
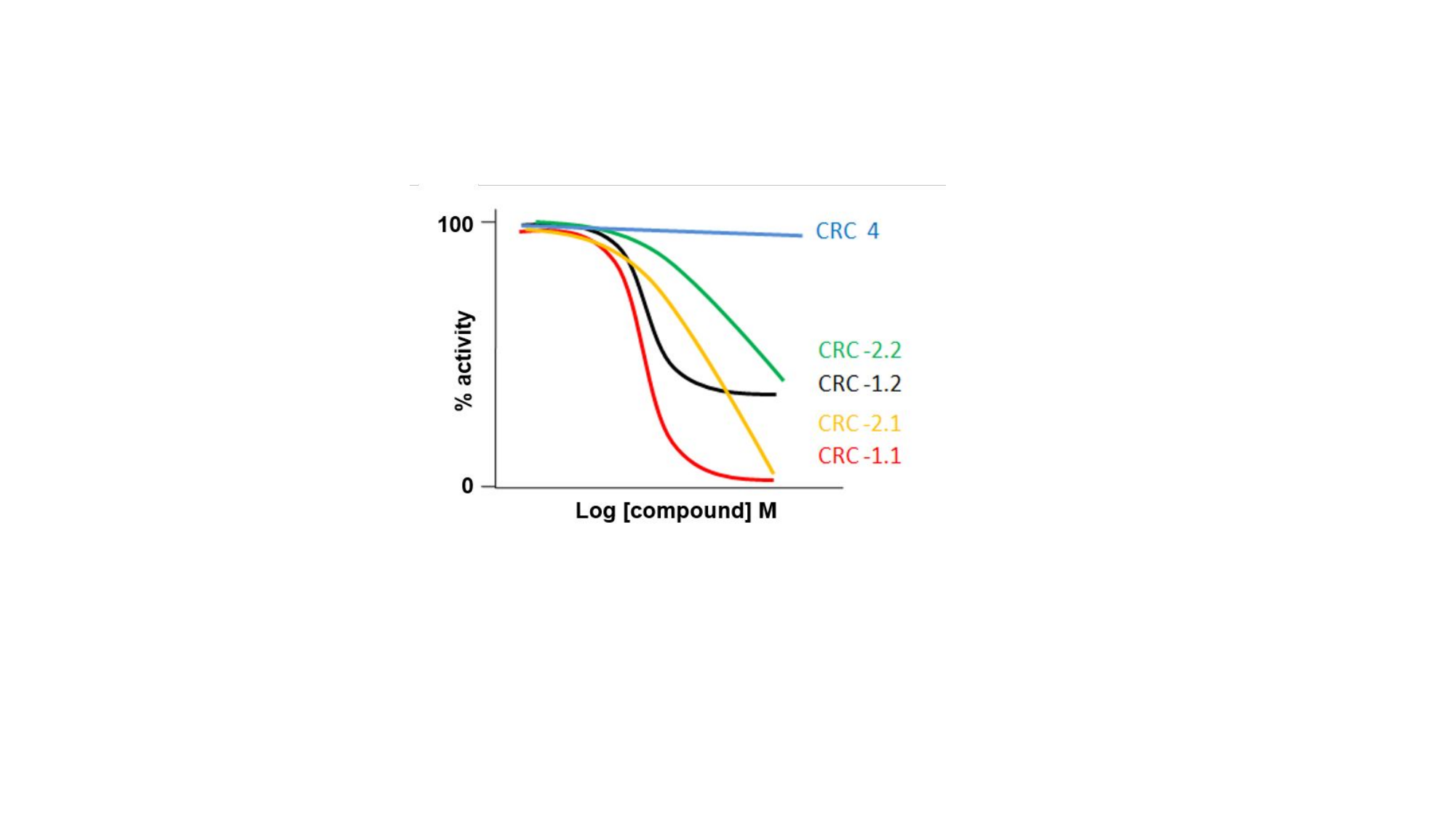

Supplement: Supplementary file 6 — Additional file 6: Figure S4. Diagrams of different dose responses and the corresponding curve response class (CRC) scores. CRC parameters integrate potency and efficacy measurements of the compounds. Drug response curves with CRC −1.1 exhibit a near complete maximum response; −1.2 exhibit less effective maximum cell killing; −2.1 do not have a maximum response but can achieve killing of nearly all the cells; −2.2 do not have a maximum response and can only achieve intermediate killing; and 4 are inactive. [file 12967_2017_1197_MOESM6_ESM.pptx]

## Slide 1
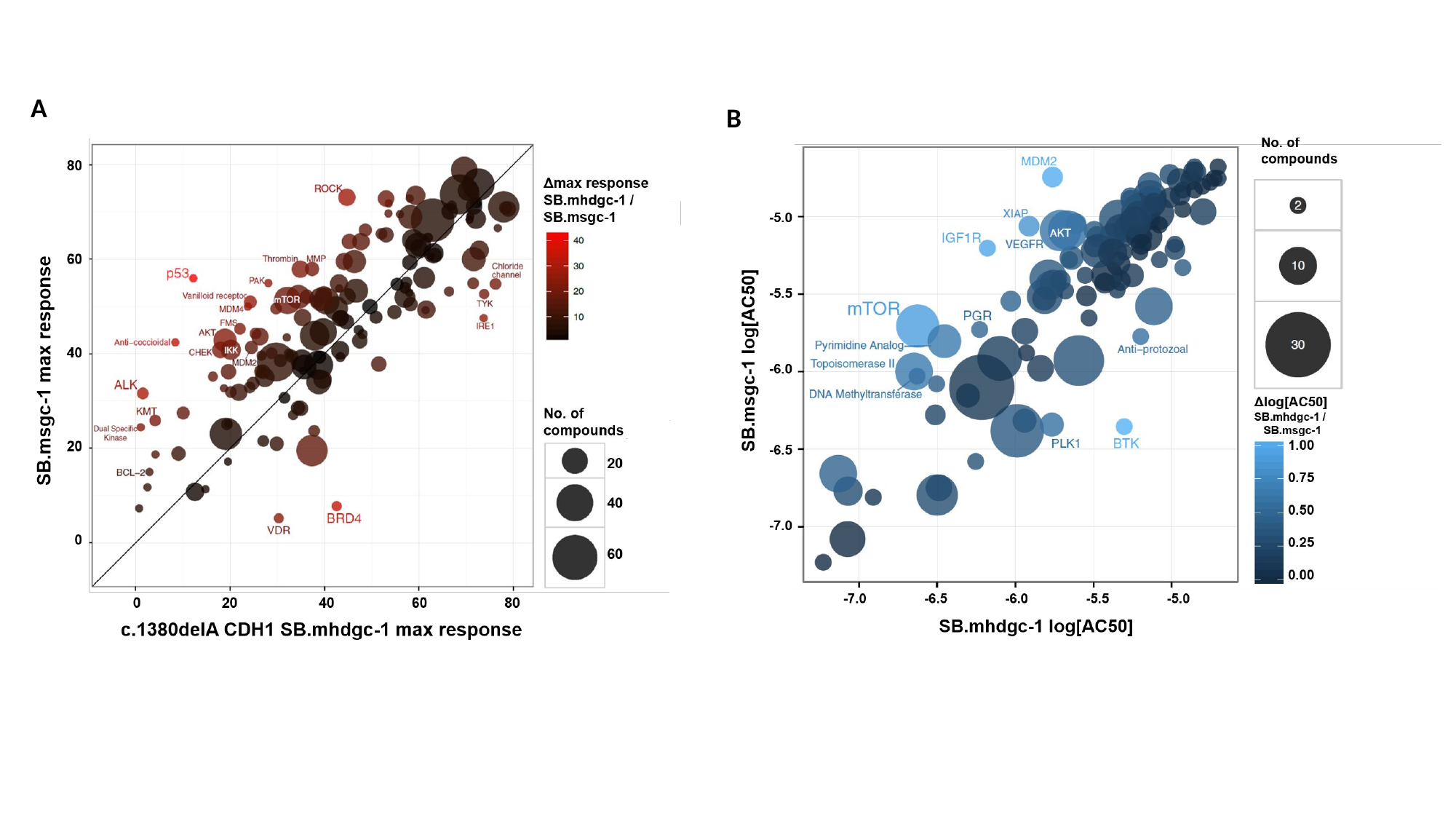

A
B

Supplement: Supplementary file 7 — Additional file 7: Figure S5. Selective pharmacological vulnerabilities of c.1380delA SB.mhdgc-1 versus SB.msgc-1 gastric cancer cells identified by comparative qHTS screening with the MIPE Oncology 4.0 library. A, Bubble diagram of drug phenotypes by compound class of SB.mhdgc-1 versus SB.msgc-1 cells depicting class activities (number of compounds per drug class) measured by maximum response (max response SB.mhdgc-1 / max response SB.msgc-1). B, Bubble diagram comparing drug activities measured by potency (logAC50SB.mhdgc-1 versus logAC50SB.msgc-1). [file 12967_2017_1197_MOESM7_ESM.pptx]

## Slide 1
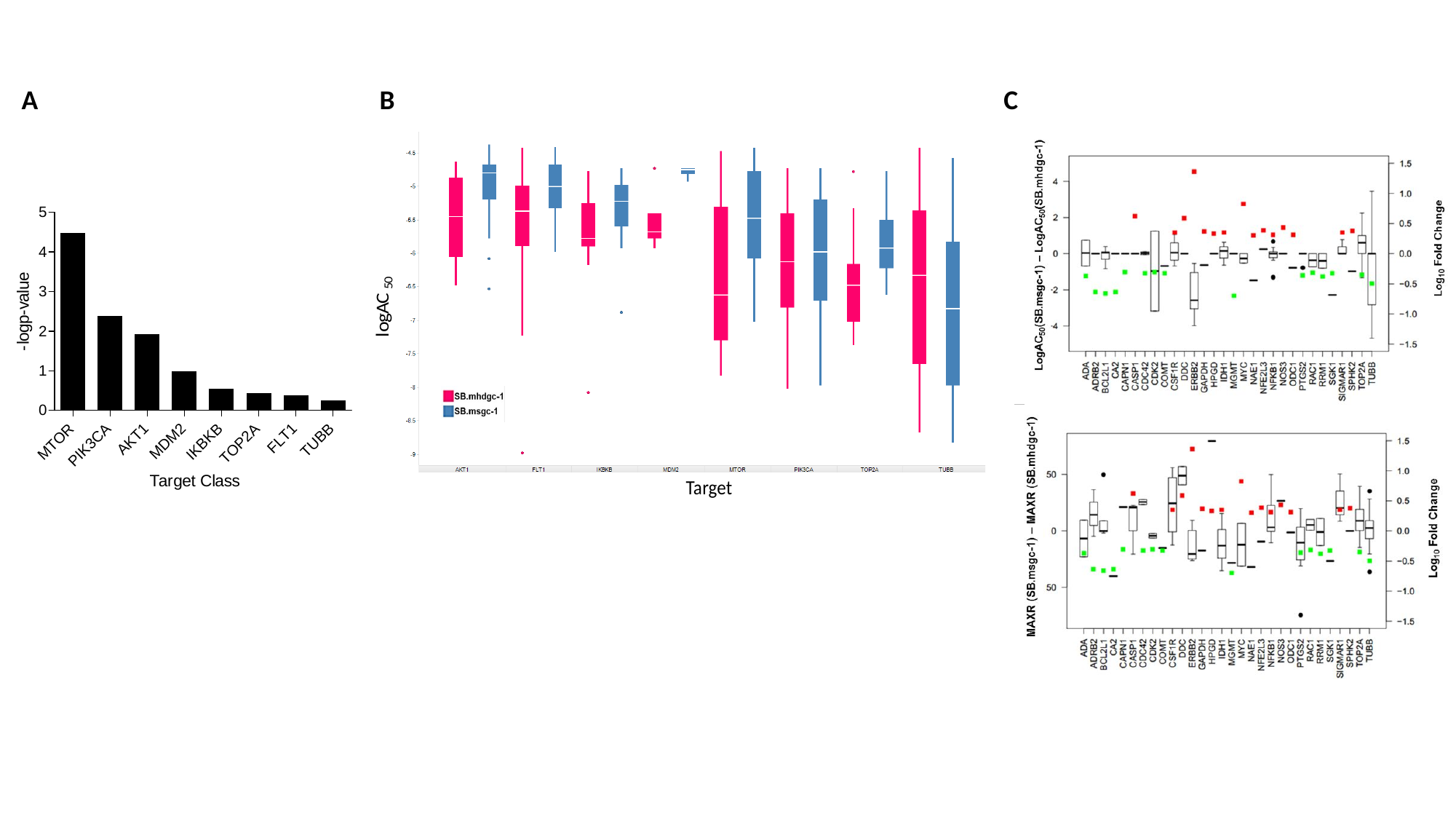

B
C
A

Supplement: Supplementary file 8 — Additional file 8: Figure S6. A, Target enrichment for compounds with CRC −1.1, −1.2, −2.1, or −2.2 and delta logAC50 (SB.mhdgc-1 logAC50–SB.msgc-1 logAC50) < −1. −log p values were calculated as described in materials and methods based on the total number of compounds targeting a gene or mechanism. B, LogAC50 distributions of compounds that show CRC −1.1, −1.2, −2.1, or −2.1 and logAC50 <−1 organized by enriched target class in c.1380delA CDH1 SB.mhdgc-1 versus SB.msgc-1 gastric cancer cells. Box plots of median logAC50 values of select target classes showing enrichment in c.1380delA CDH1 SB.mhdgc-1 (red plots) and SB.msgc-1 (blue plots). C, Concomitant dysregulation of target genes and selective activity of MIPE compounds in c.1380delA CDH1 SB.mhdgc-1 versus SB.msgc-1 gastric cancer cells. Genes with greater than two-fold expression difference in c.1380delA CDH1 SB.mhdgc-1 compared to SB.msgc-1 cells and which are direct targets of compounds in MIPE Oncology 4 are shown. Genes that are downregulated in c.1380delA CDH1 SB.mhdgc-1 compared to SB.msgc-1 cells are represented by green dots; red dots represent upregulated genes (log10 fold change indicated on the right). Overlaid are boxplots for difference in logAC50 (LAC50) SB.mhdgc-1–SB.msgc-1 for all compounds in MIPE Oncology 4 per target (top) or maximum response (bottom). [file 12967_2017_1197_MOESM8_ESM.pptx]

## Slide 1
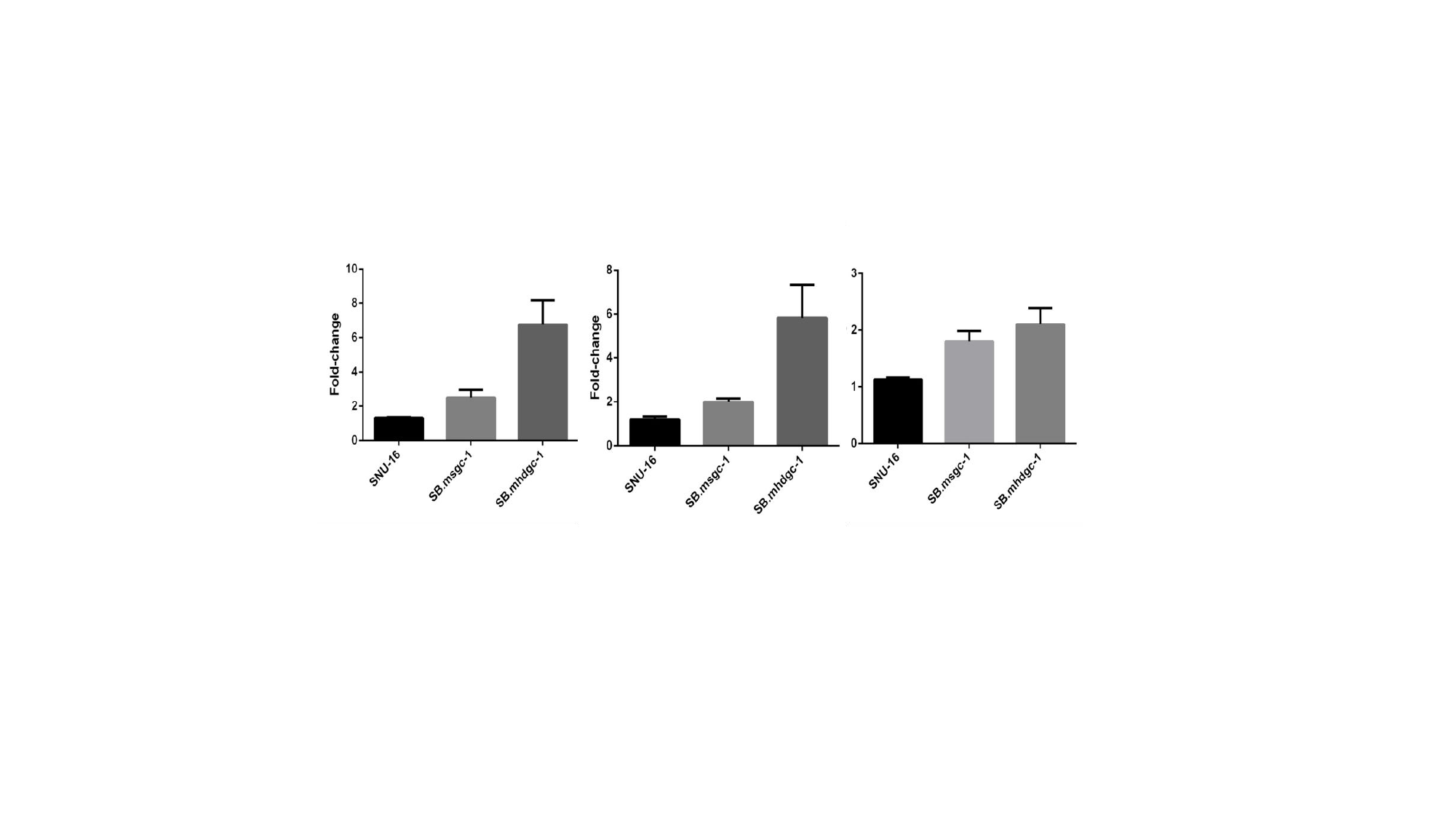

Supplement: Supplementary file 9 — Additional file 9: Figure S7. Increased induced cell death upon treatment with topoisomerase II and dual PI3K/mTOR in hereditary c.1380delA CDH1 SB.mhdgc-1 versus sporadic gastric cancer cell lines. Caspase 3/7 levels after 24 h of treatment with 1 µM mitoxantrone, 1 µM etoposide, or 1 µM PI-103 normalized to DMSO-treated control is shown (standard errors of the mean from at least 2 independent experiments done in triplicate shown). [file 12967_2017_1197_MOESM9_ESM.pptx]
